# In situ snapshots along a mammalian selective autophagy pathway

**DOI:** 10.1073/pnas.2221712120

**Published:** 2023-03-14

**Authors:** Meijing Li, Ishita Tripathi-Giesgen, Brenda A. Schulman, Wolfgang Baumeister, Florian Wilfling

**Affiliations:** ^a^Department of Molecular Structural Biology, Max Planck Institute of Biochemistry, 82152 Martinsried, Germany; ^b^Department of Molecular Machines and Signaling, Max Planck Institute of Biochemistry, 82152 Martinsried, Germany; ^c^Mechanisms of Cellular Quality Control, Max Planck Institute of Biophysics, 60438 Frankfurt a. M., Germany

**Keywords:** cryo-electron t​omo​gra​phy​, xenophagy, autophagosome, *Salmonella*, omegasome

## Abstract

Selective macroautophagy (hereafter referred to as autophagy) describes a process in which cytosolic material is engulfed in a double membrane organelle called an autophagosome. Autophagosomes are carriers responsible for delivering their content to a lytic compartment for destruction. The cargo can be of diverse origin, ranging from macromolecular complexes to protein aggregates, organelles, and even invading pathogens. Each cargo is unique in composition and size, presenting different challenges to autophagosome biogenesis. Among the largest cargoes targeted by the autophagy machinery are intracellular bacteria, which can, in the case of *Salmonella,* range from 2 to 5 μm in length and 0.5 to 1.5 μm in width. How phagophores form and expand on such a large cargo remains mechanistically unclear. Here, we used HeLa cells infected with an auxotrophic *Salmonella* to study the process of phagophore biogenesis using in situ correlative cryo-ET. We show that host cells generate multiple phagophores at the site of damaged *Salmonella*-containing vacuoles (SCVs). The observed double membrane structures range from disk-shaped to expanded cup-shaped phagophores, which have a thin intermembrane lumen with a dilating rim region and expand using the SCV, the outer membrane of *Salmonella*, or existing phagophores as templates. Phagophore rims establish different forms of contact with the endoplasmic reticulum (ER) via structurally distinct molecular entities for membrane formation and expansion. Early omegasomes correlated with the marker Double-FYVE domain-Containing Protein 1 (DFCP1) are observed in close association with the ER without apparent membrane continuity. Our study provides insights into the formation of phagophores around one of the largest selective cargoes.

## Significance

Eliminating intracellular pathogens by selective autophagy, termed xenophagy, is an important defense mechanism ensuring cellular integrity during viral and bacterial infections. Malfunction of xenophagy is associated with an increased number and persistence of intracellular pathogens. To engulf whole bacteria, phagophore membranes expand dramatically. The process of their formation and expansion is still elusive. We used correlative cryo-electron tomography (cryo-ET) to investigate phagophore formation during *Salmonella* clearance in situ. The tomograms reveal insights into the organization and appearance of xenophagic structures, including omegasome-like structures, phagophores, and various contact sites between the phagophore rim and the endoplasmic reticulum (ER). These results open avenues to study the mechanisms of autophagosome biogenesis for one of the largest autophagic cargoes.

Autophagy is a conserved cellular pathway for the degradation of cytosolic components, such as protein aggregates (aggrephagy), membrane-bound organelles, and intracellular pathogens (xenophagy). Xenophagy specifically eliminates intracellular pathogens such as viruses and bacteria and, as such, is an essential component of the innate immune system (1, 2). *Salmonella*, a severe disease-causing gram-negative bacterium, is cleared by xenophagy in a dynamic process. Upon entry into the host cell, *Salmonella* initially resides in the SCV, which enriches the small GTPase RAB5B and the lysosomal-associated membrane protein 1 (LAMP1) (3). For replication, a fraction of *Salmonella* breaks out of the SCV; rupture of the SCV membrane is recognized by galectin-8 (GAL8), which leads to mammalian target of rapamycin (mTOR) inactivation and hence activation of autophagy (4, 5). In addition, cytosol-exposed *Salmonellae* are polyubiquitylated and recognized by ubiquitin-dependent autophagy receptors (6, 7). Interaction of the receptors with the autophagy machinery targets the machinery to the *Salmonella* surface to initiate the formation of the cup-shaped, double-membrane phagophore; the phagophore expands and closes to form an autophagosome that engulfs the *Salmonella*. Finally, mature autophagosomes fuse with lysosomes to degrade the engulfed bacteria (8).

A critical feature of this process is the initiation and expansion of the phagophore membrane. Extensive studies on phagophore formation have focused on amino acid starvation-induced autophagy. Fluorescent microscopy (9) and immuno-transmission electron microscopy (TEM) (10–12) provide evidence that a PI3P-rich subdomain of the endoplasmic reticulum (ER) transforms into a phagophore referred to as the omegasome. Additionally, autophagy-related protein 9 (ATG9)-positive compartments are thought to form seeds for phagophore membrane formation (13, 14). Phagophores initiated from omegasomes and ATG9 vesicles need phospholipids to expand the phagophore membrane and ultimately encapsulate the cytosolic cargo (15). Generally, three routes have been described for the delivery of phospholipids for membrane expansion: 1) protein-mediated direct lipid transfer, 2) vesicle-mediated membrane fusion, and 3) extrusion of membranes from preexisting organelles (16). The direct lipid transfer pathway assembles newly synthesized phospholipids at the ER into the phagophore membrane via lipid transfer proteins (LTPs) (17). The lipid transfer complex ATG2/WIPI4 (ATG2/ATG18 in budding yeast) has been suggested to transfer lipids from the ER to the phagophore membrane (18, 19). However, other lipid transfer proteins, such as VPS13, are thought to play a similar role in this process (20). In line with this, conventional TEM studies have observed close proximity between ER and phagophores (10, 11, 21, 22). In addition, ER-Golgi intermediate compartment-derived COPII vesicles serve as templates for mammalian microtubule-associated protein 1 light chain 3 (MAP1-LC3, or LC3) lipidation, which feeds phagophore expansion (23, 24). Mitochondria (25), endosomes (26), Golgi (27), plasma membrane (28), and lipid droplets (29) have also been reported to play a role as membrane sources for the formation and growth of phagophores.

A structural understanding of autophagosome formation during xenophagy is still missing. Intracellular bacteria are not only one of the largest autophagic cargoes, but the process is also turned on by several autophagy initiators immediately and directly upon their exposure to the cytosol. As such, they are an excellent model system for studying autophagosome biogenesis during selective autophagy. Here, we used in situ correlative cryo-ET in combination with cryo-focused ion beam milling (30) to study the ultrastructure of phagophores engulfing *aroA*-deficient *Salmonella enterica serovar* Typhimurium (hereafter *Salmonella*). We provide high-resolution data on *Salmonella* clearance by xenophagy in HeLa cells at different stages of the process. We show that host cells generate multiple phagophores around *Salmonella*, ranging from biconcave disk-shaped to expanded phagophores. The SCV, the outer membrane of *Salmonella*, and the existing phagophore serve as templates for phagophore growth. Correlated DFCP1 signals frequently occur at several sites around *Salmonella,* where phagophores exhibit features of omegasomes. Finally, our data reveal that the phagophore rim provides an interactive platform for establishing multiple connections to the ER, either through stick-shaped densities or macromolecular clusters. Collectively, our work provides an ultrastructural characterization of a selective mammalian autophagy pathway.

## Results

### Characterization of SCV Rupture during Infection.

**Fig. 1.**
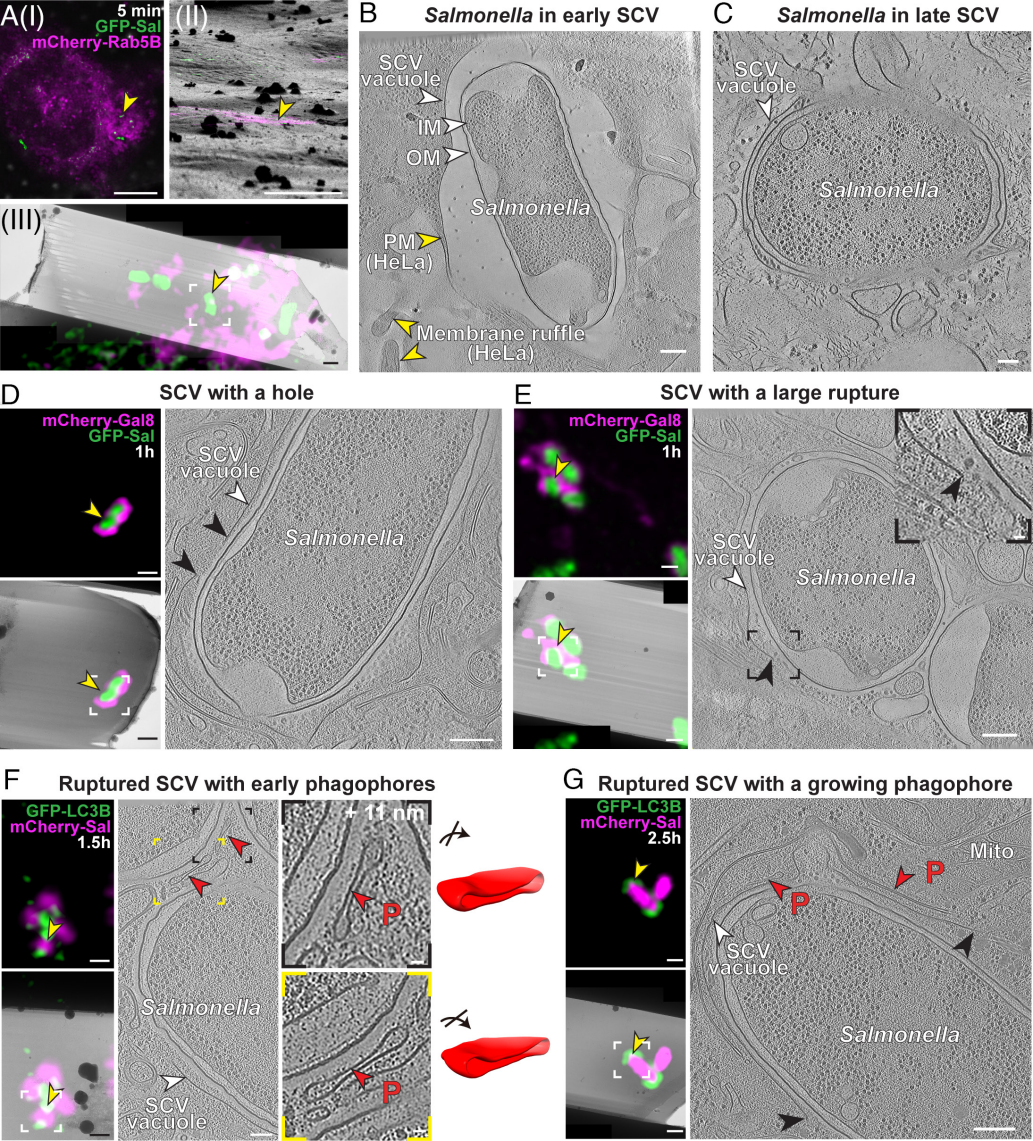
SCV damage triggers phagophore formation and expansion. (*A*) Steps of the correlative cryo-ET workflow, including cryo-fluorescent imaging (*i*), 3-dimensional (3D) correlation of the fluorescent signal to the SEM image (*ii*), and 2-dimensional (2D) correlation of the fluorescent signal to the TEM obtained lamella map (*iii*). Yellow arrowheads indicate the targeted *Salmonella*. The white box indicates the cryo-ET tilt series acquisition area. (*B* and *C*) mCherry-RAB5B-expressing HeLa cells were infected with GFP*-*expressing *Salmonellae*. Tomographic slices show *Salmonella* in different intact SCVs at 5 min p.i.; See *SI Appendix*, Fig. S1*A* for the correlative overviews of (*C*). (*D* and *E*) Correlative cryo-ET at 1 h p.i. reveals damage to the SCV. Black arrowheads indicate the ruptured fragments of SCV vacuoles. (*F* and *G*) GFP-LC3B-expressing HeLa cells were infected with mCherry-expressing *Salmonellae*. Correlative cryo-ET captures early phagophores (*F*) and expanded phagophores (*G*) around a damaged SCV. Red arrowheads indicate phagophores around ruptured SCVs. The scale bars represent 10 µm in (*A* (*i*), (*ii*)), 1 µm in (*A* (*iii*), the *Left* panels of *D*, *E* *F*, and *G*), 100 nm in (*B*, *C*, the *right* panels of *D*, *E*, *F (middle)*, and *G*), 20 nm in enlarged views in *E* and *F*. Yellow arrows and white boxes in the overview images show targeted *Salmonella* and areas of tomogram acquisition, respectively. PM, plasma membrane of the HeLa cell; IM, *Salmonella* inner membrane; OM, *Salmonella* outer membrane; P, phagophore.

Rupture of the SCV membrane exposes the *Salmonella* surface to the cytosol, triggering a selective autophagy response (4, 31). To distinguish differences in SCV integrity, we generated stable HeLa cells expressing different fluorescently tagged proteins that mark different stages after *Salmonella* infection. At different time points postinfection (p.i.), the cells were plunge frozen before being subjected to a correlative cryo-ET workflow (30) (Fig. 1*A*). Guided by the mCherry-tagged early endosomal marker RAB5B at 5 min p.i., the workflow revealed *Salmonellae* encapsulated in SCVs near the host plasma membrane (PM), as indicated by PM ruffles (Fig. 1*B*), as well as at later stages within the host cytosol (*SI Appendix*, Fig. S1*A* and Fig. 1*C*). With time, the SCVs start getting more compact, leading to a tighter spacing between the outer *Salmonella* membrane (OM) and the SCV membrane (Fig. 1 *B* and *C*). This enables the type-III secretion system to perforate the membrane of the SCV and inject pathogenic factors into the host cytosol (31) (Fig. 1 *C*). A loss of SCV integrity exposes the *Salmonella* surface and glycosylated proteins within the SCV membrane. These exposed glycosylated proteins are recognised by the cytosolic galectin GAL8 and accumulation of mCherry-GAL8 therefore serves as a marker for SCV membrane damage (Fig. 1 *D* and *E*). Tomographic analysis at correlation sites showed that SCV membrane rupture could range from holes within the SCV membrane (Fig. 1 *D* and *E*) to almost a complete loss of the membrane (*SI Appendix*, Fig. S1*B*). In addition, double-membrane structures resembling potential autophagic intermediates were observed after SCV rupture (Fig. 1*D* and *SI Appendix*, Fig. S1*B*). To confirm that these double-membrane structures resembled phagophores, we expressed a green fluorescent protein (GFP)-tagged version of the autophagy marker LC3B (Fig. 1 *E* and *F*). As expected, the LC3B signal corresponded to phagophores at various maturation stages around cytosolically exposed *Salmonella* cells (Fig. 1 *F* and *G*). Early phagophores appeared as biconcave disks with granular electron densities in the intermembrane volume of the dilated rim region (Fig. 1*F*), suggesting the potential existence of proteins inside the phagophore lumen.

### SCV Rupture Triggers the Formation of Multiple Phagophores at the *Salmonella* Surface.

**Fig. 2.**
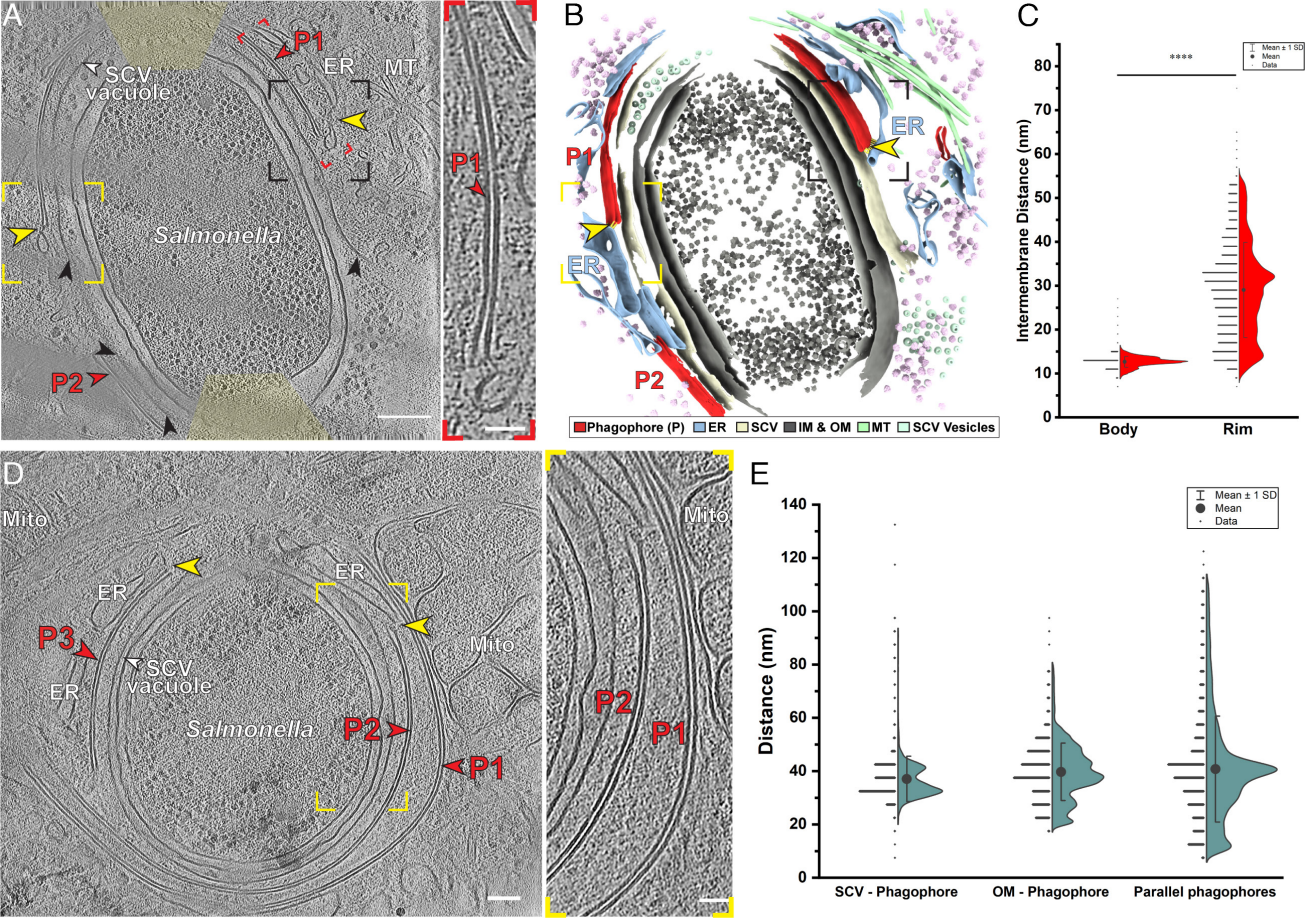
Characterization and organization of phagophores during selective autophagy. (*A*) Tomographic slice of a damaged SCV targeted by two expanded phagophores (P1 and P2). Black arrowheads indicate the ruptured fragments of the SCV vacuole. See also Movie S1. The mCherry-GAL8-expressing HeLa cells were infected with GFP*-*expressing *Salmonellae* and analyzed at 1.5 h p.i. (*B*) 3D rendering of features in (*A*). (*C*) Violin plot showing the intermembrane distances of the phagophore body and rim region. 75 rims of 53 expanded phagophores were analyzed. The average distances are 12.6 ± 1.2 nm and 29.0 ± 10.9 nm, respectively. Distances were calculated from membrane middle to membrane middle. (*D*) The tomographic slice highlights a phagophore's expansion (P1) on top of the existing phagophores (P2). The enlarged view highlights the distance between P1 and P2. The mCherry-GAL8-expressing HeLa cells were infected with GFP*-*expressing *Salmonellae* and analyzed at 1.5 h p.i. (*E*) The violin plot shows the distances of phagophores to the SCV (N = 48 phagophores), *Salmonella* OM (N = 53 phagophores), or another phagophore (N = 12 phagophores). The mean distances are 39.7 ± 10.7 nm, 37.0 ± 8.5 nm, and 40.8 ± 19.9 nm, respectively. Yellow arrowheads indicate contact sites between the phagophore rim and the ER. The scale bars represent 100 nm (*A* and *D*) and 50 nm (*A* and *D* *Insets*).

Our previous cryo-ET analysis of phagophores in *Saccharomyces cerevisiae* revealed a narrow intermembrane distance (10.6 ± 0.9 nm) different from other double-membrane organelles. Moreover, it showed a dilated rim structure with a wider intermembrane spacing compared to the rest of the phagophore (32). Both features were also observed for expanded phagophores during xenophagy (Figs. 1*G* and 2 *A* and *B* and *SI Appendix*, Fig. S2). On average, the intermembrane spacing was found to be 12.6 ± 1.2 nm for the phagophore body and 29.0 ± 10.9 nm for the dilated rim area (Fig. 2*C*). Of note, the observed rim dilation was, on average, more pronounced than that in *S. cerevisiae* (*SI Appendix*, Fig. S2 *A* and *C*). This is in line with previous reports, which show similar dilation phenotypes of the phagophore rim for other selective forms of autophagy in mammalian cells (21).

Interestingly, multiple phagophores often formed at the same *Salmonella* (Fig. 2 *A* and *D* and Movie S1). The phagophores grew either around the SCV (Fig. 2 *A* and *B*, *SI Appendix*, Fig. S2*A*, and Movie S1), the *Salmonella* OM (*SI Appendix*, Fig. S2*B*), or in some cases, on the top of existing phagophores. (Fig. 2*D*). The initiation of multiple phagophores was independent of LC3B overexpression since all described cases were also observed in mCherry-GAL8 overexpressing cell lines (Fig. 2 *A* and *D* and *SI Appendix*, Fig. S2). Many of the individual phagophores were in close proximity to the ER, suggesting the simultaneous expansion of multiple phagophores (Fig. 2 *A*, *B*, and *D* and *SI Appendix*, Fig. S2*B*; indicated by yellow arrows). The observation that phagophores can expand on top of each other also explains the onion-like structures seen in previous studies (33).

To understand the relationship between the cargo-phagophore interface, we analyzed the corresponding distances. Notably, phagophores were found exclusively around the cargo surface in the tomographic volumes analyzed (Fig. 2 *A*, *B*, and *D* and *SI Appendix*, Fig. S2 *A* and *B*). On average, the distance between the cargo surface and the phagophore membrane was 39.7 ± 10.7 nm for the SCV and 37.0 ± 8.5 nm for the OM (Fig. 2*E*). A larger range for the distance was observed when another phagophore served as a template (40.8 ± 19.9 nm) (Fig. 2 *E* and *D*, *Inset*). In one case, we observed a *Salmonella* with a phagophore encapsulated within a single autophagosome, suggesting that expansion of the inner phagophore was unsuccessful (*SI Appendix*, Fig. S2*C* and Movie S2). In an extreme case, we even found two *Salmonella* cells surrounded by a giant phagophore that sequestered two phagophores, host ribosomes, and vesicles, thereby losing the cargo exclusivity (*SI Appendix*, Fig. S2*D*). Taken together, our data show that multiple phagophores can be initiated on a single *Salmonella* after SCV rupture and that the *Salmonella* surface is not the only cargo template for phagophore expansion.

### The Phagophore Rim Establishes Membrane Contact Sites to the ER via Various Macromolecules.

**Fig. 3.**
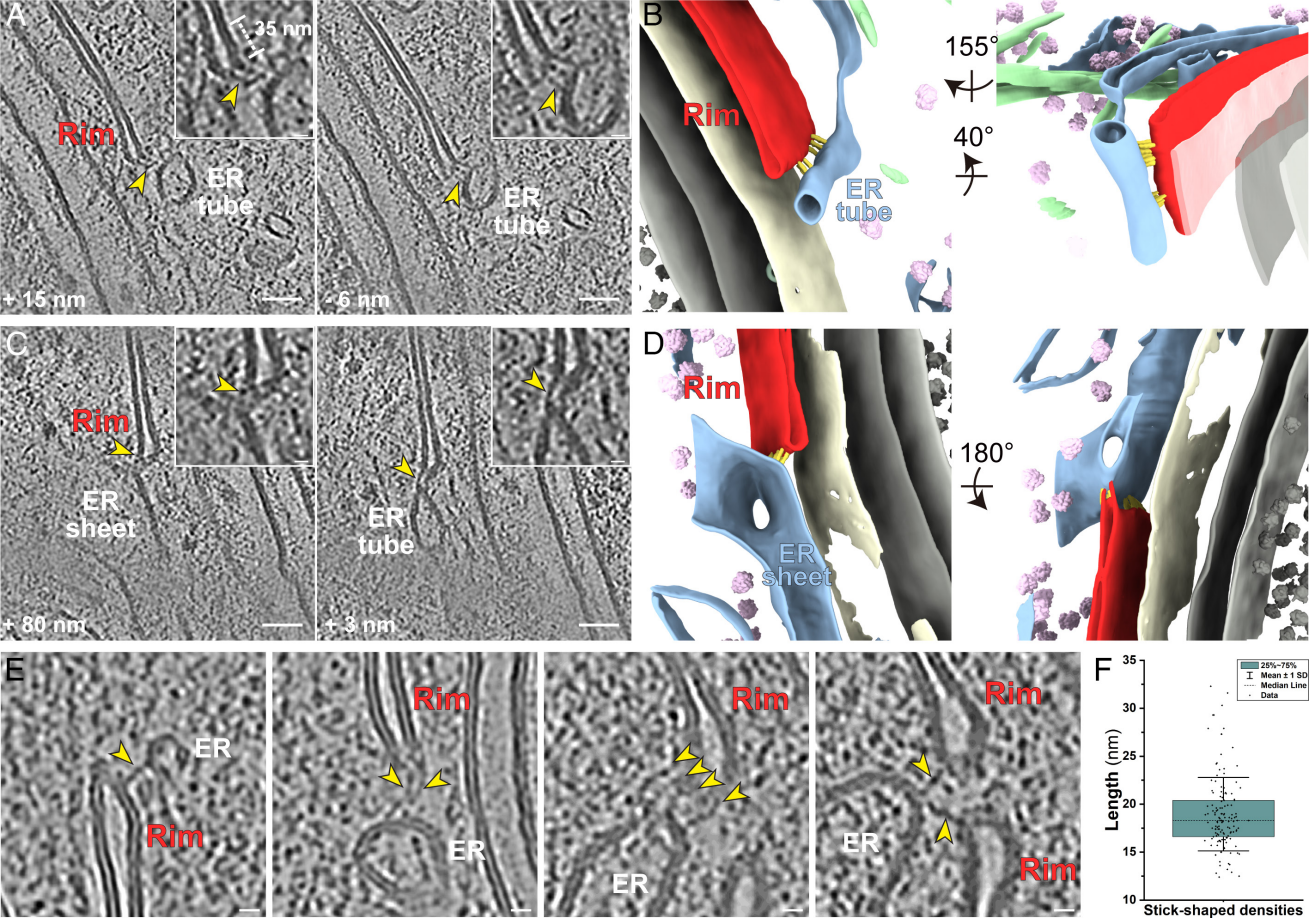
Expanding phagophores contact the ER via stick-shaped densities. (*A*) Enlarged tomographic slices marked by the black box in Fig. 2*B* show that the phagophore rim connects with an ER tube via stick-shaped proteins (indicated by yellow arrowheads). (*B*) 3D rendering model of features in (*A*). (*C*) Enlarged tomographic slices marked by the yellow box in Fig. 2*B* also show that the other phagophore rim connects with an ER sheet and tube through the stick-shaped densities (indicated by yellow arrowheads). (*D*) 3D rendering model of features in (*C*). (*E*) Tomogram slices show different forms of stick-shaped densities at phagophore rim–ER contact sites. (*F*) The average length of the stick-shaped densities is 19.0 ± 3.9 nm (N = 138, from 16 rims). The scale bars represent 50 nm in (A and *C*) and 10 nm in *E*.

The expansion of the phagophore membrane during starvation-induced autophagy is mediated by lipid transfer proteins such as the ATG2/WIPI4 complex (18) and additionally VPS13 in budding yeast (20). Both complexes transfer phospholipids from the ER membrane to the phagophore rim (20). As expected, membrane contact sites between the phagophore rim and the ER were frequently observed, in precisely 43 out of 75 rims (Fig. 3 *A*–*D*). These areas were often occupied by stick-shaped densities, connecting the membranes of both compartments (Fig. 3 *A*–*E* and Movies S3 and S4). On average, these densities span a distance of 19.0 ± 3.9 nm with a width of approximately 4 nm (Fig. 3*F*). Interestingly, stick-shaped densities were also observed between thin tubular vesicles and the phagophore rim (*SI Appendix*, Fig. S3).

**Fig. 4.**
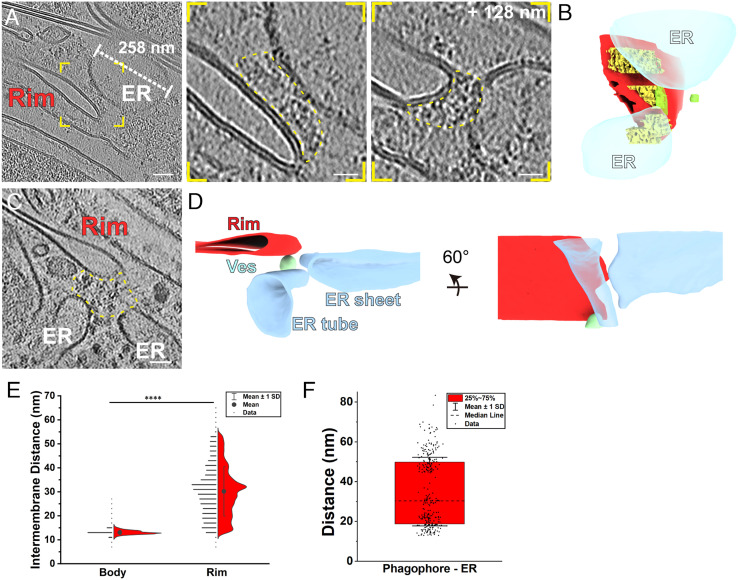
The phagophore rim can also form extended contact sites to the ER via macromolecule clusters. (*A*) Tomographic slices show a pronounced dilated rim associated simultaneously with two ER sites through macromolecule clusters (indicated by the electron densities within the area of the yellow dotted line). The mCherry-GAL8-expressing HeLa cells were infected with GFP*-*expressing *Salmonellae* and analyzed at 1.5 h p.i. (*B*) 3D rendering models of the phagophore rim in (*A*). (*C*) The tomographic slice shows another dilated rim associated simultaneously with an ER tube and ER sheet through macromolecule clusters (indicated by the electron densities within the area of the yellow dotted line). The mCherry-GAL8-expressing HeLa cells were infected with GFP*-*expressing *Salmonellae* and analyzed at 1.5 h p.i. (*D*) 3D rendering models of the phagophore rim in (*C*). (*E*) Violin plot showing the intermembrane distance of phagophores (body and rim) establishing ER contacts via macromolecule clusters (from four phagophores, six rims). The mean intermembrane distance is 12.7 ± 0.7 nm and 27.8 ± 12.1 nm, respectively. (*F*) Violin plot showing the distance between phagophore rim and ER (the same phagophores were analyzed as in (E)). The mean distance is 50.5 ± 10.6 nm. Scale bars represent 50 nm in (*A* and *C*) and 20 nm in the enlarged images of (*A*).

In addition to the stick-shaped densities, we observed two ER–rim contact sites occupied by an electron-dense material between the two membrane compartments (Fig. 4 *A*–*C* and Movie S5). In both cases, the ER–rim contact sites were established at highly dilated phagophore rim structures (Fig. 4 *A*–*E*). On average, the material filled a distance of approximately 50.5 ± 10.6 nm (Fig. 4*F*), larger than the distance observed for the first type of ER–rim contact sites.

Analysis of the phagophore rim connections highlights the establishment of different ER–rim contact sites which contain stick-shaped densities or macromolecule clusters. Although the length of the stick-shaped densities resembles those seen for Atg2 (~16 nm) (34–36) and Vps13 (~16 nm) (37), their identities remain to be further investigated.

### DFCP1-Positive Structures Reveal Insight into Omegasome Architecture.

**Fig. 5.**
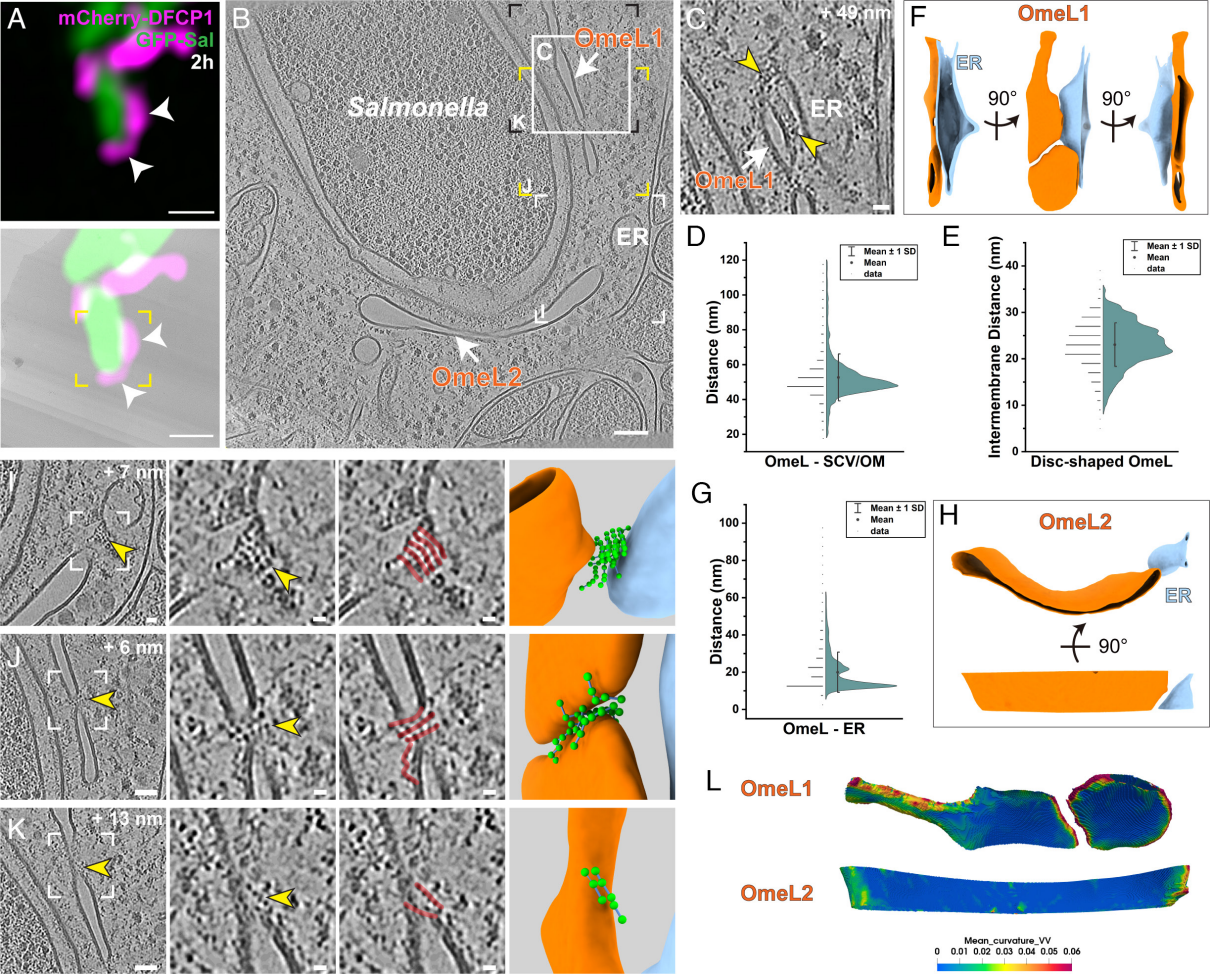
Analysis of DFCP1-positive structures. (*A*) Cryo-fluorescent image and the corresponding lamella overlay show two positions with mCherry-DFCP1 signal around a *Salmonella*. The mCherry-DFCP1-expressing HeLa cells were imaged at 2 h p.i. White arrowheads indicate the targeted DFCP1 signals. (*B*) The tomographic slice corresponding to the position in (*A*) shows two omegasome-like structures (OmeL1 and OmeL2) around *Salmonella*. (*C*) The enlarged tomographic slice of (*B*) highlights that OmeL1 is parallel to the ER and connected by electron-dense structures (indicated by yellow arrowheads). (*D*) Violin plot showing the distance between omegasomes and SCV vacuole or outer membrane of *Salmonella* with an average distance of 52.7 ± 13.5 nm (N = 8). (*E*) Violin plot showing the distribution of the intermembrane distance of the disc-shaped omegasomes (N = 6). The mean intermembrane distance is 23.1 ± 4.7 nm. (*F*) 3D rendering model of OmeL1 and the ER. (*G*) Violin plot showing the distance between the disc-shaped omegasomes and the ER. The mean distance is 20.0 ± 10.8 nm (N = 6). (*H*) 3D rendering model of OmeL2 and the ER. (*I*) Sequential tomographic slices and 3D rendering model show the filamentous densities between the ER tube and the omegasome. (*J* and *K*) Sequential tomographic slices and 3D rendering models show the filamentous densities between the neighboring omegasome discs. (*L*) 3D visualization of the curvedness of two omegasome-like structures. OmeL, omegasome-like structure. Scale bars represent 1 µm in (*A*), 100 nm in (*B*), and 20 nm in (*C*, *I*, *J*, and *K*).

In mammalian autophagy, phagophores originate from specific sites that are closely associated with subdomains of the ER. These domains are enriched in the lipid phosphatidylinositol 3-phosphate (PI(3)P) and referred to as omegasomes (9, 10). DFCP1 contains an ER-targeting domain and two FYVE motifs able to bind PI(3)P and, as such, marks sites of omegasome formation (9). Upon starvation, DFCP1 accumulates in punctate structures in proximity of the ER. These DFCP1-positive structures grow and form rings around cargo that collapse back into a punctate structure before completely disappearing (9, 38). To capture early events during phagophore formation, we overexpressed mCherry-tagged DFCP1 and recorded 25 tomograms at sites of colocalization with the GFP-*Salmonella* signal. In line with previous reports (9), several mCherry-DFCP1 signals occur concomitantly at a single *Salmonella*. As expected for the DFCP1 correlation, we observed phagophores at various stages during autophagosome formation (Fig. 5 *A* and *B*, Movie S6, and *SI Appendix*, Figs. S4 and S5).

Particularly striking were distinct double-membrane structures. These along with expanded phagophores were among the most frequently observed structures and differ from the early phagophores described before (Fig. 5 *B* and *C* and *SI Appendix*, Fig. S4). These particular structures showed an ER-like appearance but, in contrast to the ER, have an electron-transparent intermembrane volume (Fig. 5 *B* and *C* and *SI Appendix*, Fig. S4), a typical feature of autophagic structures. They were located at an average distance of 52.7 ± 13.5 nm from the damaged SCV or the *Salmonella* OM (Fig. 5*D*), similar to phagophores correlated with EGFP-LC3B and mCherry-GAL8 (Fig. 2*E*). In previous studies, omegasomes were defined by their omega shape (9). However, the limited tomographic volume did not allow us to visualize the typical cradle shape of the omegasome. Therefore, we called these structures omegasome-like.

On average, the intermembrane distance of these omegasome-like structures was 23.1 ± 4.7 nm, which is larger than that for expanded phagophores (Fig. 5*E* and *SI Appendix*, Fig. S5*F*). Compared to early phagophores correlated by EGFP-LC3B (Fig. 1*F*), these structures showed no biconcave shape and no internal granular densities, suggesting a potentially different origin or maturation process (Fig. 5*C* and *SI Appendix*, Fig. S4). Strikingly, these structures were often tightly associated to the ER with a distance of 20.0 ± 10.8 nm, as expected for omegasomes (Fig. 5 *C*, *F*, and *G* and *SI Appendix*, Fig. S4). Although no obvious membrane continuity with the proximal ER was observed within the tomographic volume, various electron-dense structures were found at the interface (Fig. 5*C* and *SI Appendix*, Fig. S4 *C*–*F*, indicated with yellow arrowheads), arguing for a particular membrane contact site between the two organelles (9, 39). However, the expanded phagophores recorded by mCherry-DFCP1 correlation were indistinguishable from phagophores correlated by EGFP-LC3B (*SI Appendix*, Fig. S5 and Fig. 1*G*) and mCherry-GAL8 (Fig. 2 *A* and *D*, *SI Appendix*, Fig. S2), suggesting that DFCP1 is located at the phagophore throughout various stages. Interestingly, in a single tomogram, an expanded omegasome-like structure formed a contact site between its dilated rim region and the ER (Fig. 5 *B* and *H*), characterized by filamentous densities (Fig. 5*I*). The filamentous densities go along the rim membrane via dot-shaped units. In the same tomogram, those densities were observed between two adjacent early omegasome-like structures (Fig. 5 *J* and *K*), at high curvature regions (Fig. 5*L*).

## Discussion

**Fig. 6.**
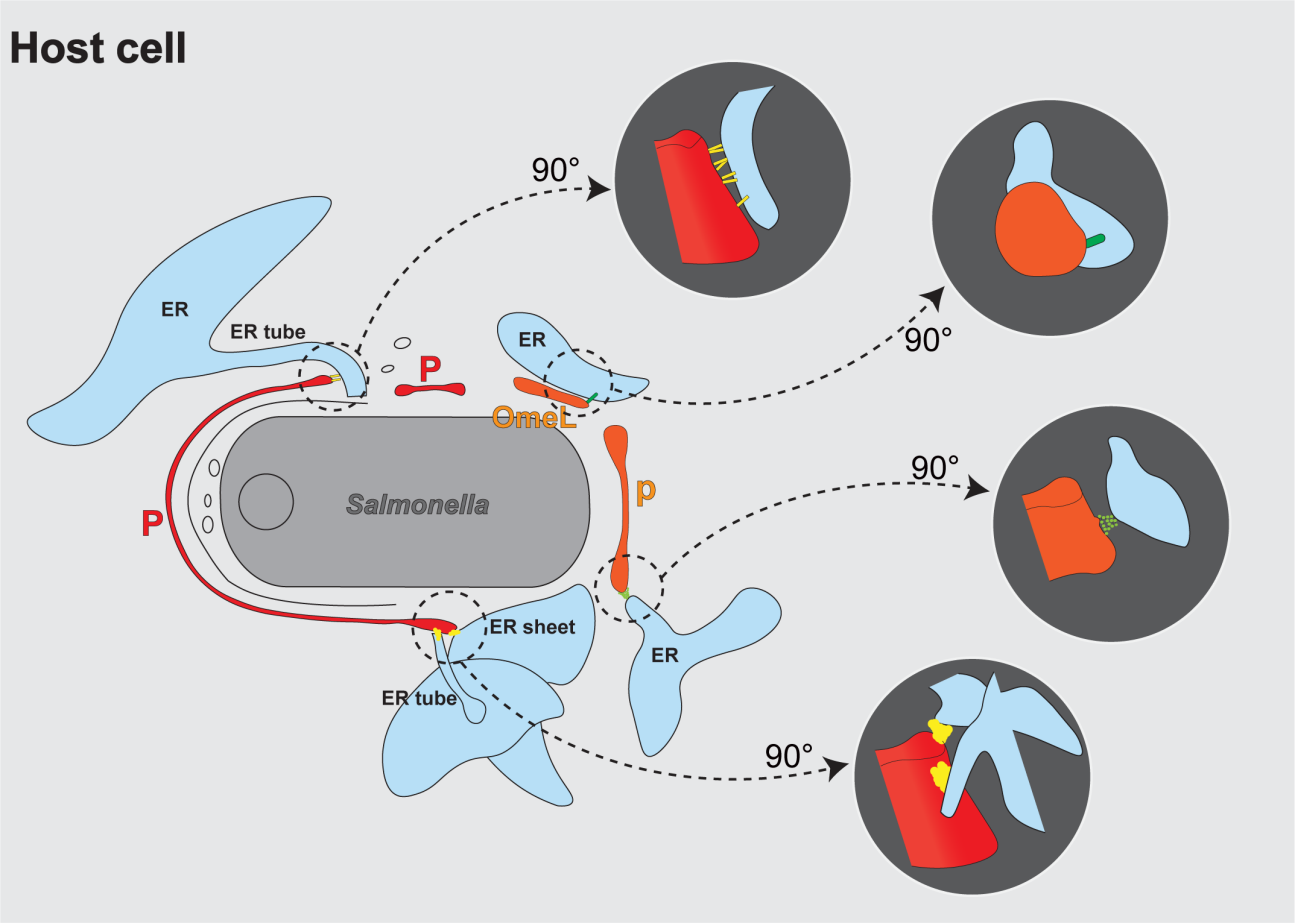
Model of phagophore formation and expansion during *Salmonella* clearance by xenophagy. The damaged SCV or cytosolic *Salmonella* triggers the formation of multiple phagophores (labeled P in red), which expand along the SCV or the outer membrane of *Salmonella*. The SCV and the outer membrane of *Salmonella* serve as templates for phagophore growth. Rim regions of the phagophore which are only slightly dilated interconnect with the ER through stick-shaped densities (colored in yellow). Some rim regions show pronounced dilation and associate with the ER through macromolecule clusters (colored in yellow). In this study, two early phagophore stages were observed. The first shows a biconcave disc-shaped structure in which the phagophore rim is significantly dilated (labeled P in red). The second type was found for sites correlated to the omegasome marker DFCP1 (labeled OmeL in orange). These omegasome-like structures were tightly associated with the ER over a large contact area. Extended omegasome-like structures (labeled P in orange) showed in rare cases a membrane connection with the ER through filamentous densities.

In this study, we used correlative cryo-ET to describe phagophore formation at sites of cytosolically exposed *Salmonella*. Analysis of the tomograms obtained provides in situ snapshots of autophagic structures at various stages, ranging from early omegasome-like to expanded phagophores (Fig. 6), allowing a glance at the structural steps underlying autophagosome formation and expansion.

Autophagy has a dual function during the *Salmonella* infection cycle. On the one hand, it protects the host cell by recognizing the cytosolically exposed bacteria and engulfing them into an autophagosome for destruction. On the other hand, it is hijacked by *Salmonella* to repair damaged SCVs (40). From the cryo-electron tomograms, it is clear that SCV damage can range from membrane holes to complete loss of the SCV membrane (41). However, even holes in the SCV membrane activate the autophagy machinery, as apparent from phagophores in the corresponding tomograms. Formation and expansion of phagophores are not only limited to the site of damage but occur along the whole SCV surface. The distance between the phagophore and the cargo is thereby maintained constant. This shows that both the bacterial OM and the SCV surface serve as templates for phagophore formation, highlighting that in most cases, the SCV membrane is engulfed alongside the bacteria. It is possible that some of the phagophores represent attempts to repair the SCV.

Frequently, multiple phagophores were observed around the same bacterium. This is in line with superresolution imaging of the ubiquitin (Ub) coat at the surface of cytosolic *Salmonella*, showing that it is not uniform but has Ub-dense and -sparse regions (42). Ubiquitylation of the SCV/bacterial surface recruits ubiquitin-dependent autophagy receptors that link cargo to the phagophore biogenesis pathway. Indeed, the distance between the phagophore surface and the SCV or bacterial surface is, on average, 40 nm. It is tempting to speculate that the reported differences in Ub signal correspond to sites of phagophore attachment.

Moreover, previous studies using conventional TEM approaches revealed multiple phagophores stacked on top of each other in an onion-like fashion (33). Our high-resolution tomograms confirmed that phagophores could grow around existing phagophores. It is conceivable that phagophores that cannot establish enough membrane contact sites for lipid transfer and expansion after their formation become a template for other expanding phagophores to ensure the completion of the autophagosome. Indeed, in some cases, phagophores at the cargo surface were trapped in an early stage and incorporated into autophagosomes (*SI Appendix*, Figs. S2*C* and S3). The factors establishing the avid interaction between phagophores for the engulfment of early unsuccessful phagophores are currently unknown.

Phagophore membrane expansion during bulk autophagy is mainly driven by lipid transfer proteins, which enrich at membrane contact sites between the phagophore and other organelles. Calculations suggest that in yeast, 60 to 80% of the required phospholipids come from direct lipid transfer. Two lipid transfer proteins have been described in the context of phagophore expansion: the ATG2/WIPI4 complex and in budding yeast additionally VPS13. Analysis of our tomographic dataset of the 65 phagophores revealed that similar to budding yeast most membrane contact sites are formed between the ER and the phagophore rim (Fig. 6) (32). Notably, some phagophores establish ER–rim contacts on only one side, suggesting that the ER–rim contact site does not necessarily span the entire phagophore rim. At the ER–rim contact site, stick-shaped densities with an average length of 19.0 ± 3.9 nm were tethered between the two membranes. This size is consistent with similarly shaped densities reported previously (17 ± 3 nm for budding yeast and ~18 nm for HeLa cells) (21, 32) and lays within the expected size range of the lipid transfer proteins ATG2 (~16 nm) (35, 36) and VPS13 (~16 nm) (37). However, the frequency with which these densities were seen at the ER–rim contact site is much higher in xenophagy than in budding yeast bulk autophagy. Whether the number of lipid transfer proteins scales with the size of the final autophagosome remains to be tested. Interestingly, in budding yeast, the number of lipid transfer proteins determines the duration and size of forming autophagosomes (20). In addition to direct lipid transfer, it is also conceivable that phagophore membranes are expanded by fusion of individual phagophores. We frequently observed multiple phagophores at various locations around *Salmonella*. Although no fusion events between phagophores were recorded, we observed a fully engulfed *Salmonella* that contained two large vesicles incorporated into the autophagosome double bilayer membrane (*SI Appendix*, Fig. S2*C*). Such a structure could result from phagophore–phagophore or phagophore–autophagosome fusion, hinting at additional mechanisms driving membrane expansion around large cargoes.

In rare cases, we observed a second type of membrane contact site between the ER and the phagophore rim, mediated by an electron-dense macromolecule cluster (Fig. 6). At these sites, multiple ER regions were connected to the same phagophore rim. Furthermore, the phagophore rim forming these contact sites showed a pronounced dilation phenotype (Figs. 4 and 6). This may reflect a distinct and transient stage during the rapid expansion of the phagophore membrane, which would explain the low frequency, but its function, as well as the factors mediating the contact, is unknown.

How is the initial phagophore formed? The role of omegasomes in autophagosome biogenesis remains controversial. While the prevailing model in budding yeast suggests that vesicle fusion plays a major role in establishing the isolation membrane, the mechanism is less clear for mammalian cells (43, 44), where both the fusion of vesicular compartments and omegasome formation have been reported to drive phagophore assembly (9). Omegasomes are thought to be membrane extensions proximal to the ER, rich in PI(3)P, from which phagophores form and are loaded with cargo. Correlation of the omegasome marker DFCP1 revealed double-membrane structures that show features of both the ER and phagophores, which we interpreted as omegasome-like structures. Yet, we observed no direct membrane continuity between the ER and the phagophore structure. FIB-milled lamellae only represent a small volume of the entire cell, and potential connections are likely to occur outside the tomographic volume (150 to 200 nm in thickness). The close association of the early DFCP1-correlated structure with the ER suggests a model in which the ER, similar to the vacuole in budding yeast, serves as a platform for the assembly of omegasome-like structures. Whether the early OmeL structures actually originate from the ER or assemble adjacent to the ER remains to be elucidated in the future. However, in the case of membrane continuity between the ER and the omegasome structure, it is still unclear how the lumen of the ER selectively retains luminal proteins. The luminal composition of OmeL structures is distinctly different from the ER lumen, resembling the empty appearance of phagophores in our tomographic analysis. Given that DFCP1-correlated structures at later stages are indistinguishable from phagophores targeted by other markers, it is likely that the early OmeL structures are a type of phagophore precursor.

Recently, DFCP1 has been shown to be an ATPase important for omegasome constriction (38). The similarities of DFCP1 to the well-studied GTPase dynamin suggest that the observed filaments at the contact sites with the ER may represent DFCP1 assemblies. These densities were seen exclusively at high curvature regions of the membrane rim. It is possible that these filaments establish or modulate membrane contact sites between the phagophore and the ER, highlighting the functional connectivity of the two organelles during autophagosome biogenesis. Nevertheless, one must interpret the observed densities and their functional relevance with caution since, in these experiments, DFCP1 was overexpressed from a cytomegalovirus promoter which might lead to altered omegasome morphology compared to endogenous expression levels.

In conclusion, we describe a tomographic dataset of a previously undescribed kind that will facilitate systematic investigations of phagophore initiation and expansion, a key step in autophagy.

## Materials and Methods

### Cell Culture, Plasmids, and Stable Line Generation.

The Flp-In™ T-REx™ HeLa cell line was a kind gift from Christian Behrends (Ludwig-Maximilians-University of Munich, Germany). HeLa cells were cultured in Dulbecco’s Modified Eagle’s Medium (DMEM) supplemented with 2 mM GlutaMAX-I, 10% fetal bovine serum, and 100 U/mL HyClone penicillin-streptomycin at 37 °C and 5% CO_2_.

GFP-LC3B (#24920), mCherry-Galectin3 (#85662), mCherry-DFCP1 (#86746), and LAMP1- mCherry (#45147) plasmids were ordered at ADDGENE. The mCherry-Galectin8 plasmid was reconstructed by replacing Galectin3 in the mCherry-Galectin3 plasmid with Galectin8, cloned from cDNA with GIBSON cloning kit. The pcDNA5-mCherry-RAB5B construct was generated by replacing LC3B in the pcDNA5-mChery-LC3B plasmid with from cDNA cloned RAB5B cDNA (GenBank: AAH32740.1).

The cells were seeded at 1 to 2 × 10^5^ per 6-well, and plasmid transfections were performed using Lipofectamine™ 3,000 transfection reagent. The fluorescent-positive cells were sorted with fluorescence-activated cell sorting (FACS) into 96-well plates. Then, the sorted single-positive cells were cultivated in a selection medium for 4 to 5 wk. The selection medium for GFP-LC3B, mCherry-DFCP1, and LAMP1- mCherry transfected cells contained G418 (Geneticin) at a concentration of 0.8 mg/mL. mCherry-Galectin8 transfected cells were selected using hygromycin (100 μg/mL) and blasticidin (15 μg/mL).

### Bacterial Culture and Infections.

*Salmonella* Typhimurium str. SL1344 *ΔaroA* strains were a kind gift from Dirk Bumann (University of Basel). *Salmonellae* were grown overnight in LB medium, with 100 mg/mL ampicillin and 25 mg/mL spectinomycin hydrochloride shaking at 220 rpm at 37 °C overnight. Then, *Salmonella* cells were subcultured (1:33) in fresh LB for 3 h to an OD_600_ of 1.2 to 1.4. *Salmonella*e were harvested by centrifugation at 12,000 g for 2 min and washed twice with an equivalent volume of antibiotic-free DMEM.

For infection, HeLa cells were seeded onto cryo-EM grids in 6-well plates (3 × 10^5^ cells/well) and cultured in antibiotic-free DMEM medium for 24 h at 37 °C in a 5% CO_2_ incubator. *Salmonella* cells in antibiotic-free DMEM medium were added onto cells at a multiplicity of infection of 150 at 37 °C and incubated for the indicated time points. Then, the cells were washed with PBS three times to remove extracellular *Salmonella* and cultured in DMEM containing 50 mg/mL gentamycin for indicated time points.

### Cryo-EM Grid Preparation.

The cryo-EM grids (200 mesh holey carbon R1/4 AU grid, Quantifoil Micro Tools) were glow-discharged to increase the hydrophilicity. After 30 min UV radiation in a laminar flow hood, the cells were seeded on the grids. Then, the prepared grids with the infected cells were subjected to plunge-freezing. For this, 4 µL blotting buffer (PBS, 10% glycerol, 0.5 mg/mL Dynabeads™ MyOne™ Carboxylic Acid) was added to the cryo-EM grids in a Vitrobot Mark IV (Thermo Fischer Scientific) with 90% humidity at 37 °C. The blot force and time were 10 and 10 s, respectively. The cryo-EM grids were stored in liquid nitrogen until use.

### Correlative Cryo-Light and Electron Microscopy and Lamella Preparation.

At first, the cryo-EM grids were imaged with a Leica SP8 cryo-confocal microscope at magnifications with an XY-pixel size of 58 to 80 nm and a z-step size of 100 nm to identify *Salmonella*-infected cells at specific autophagic stages. The Dynabeads and mCherry-labeled proteins were acquired with 552 nm laser excitation and eGFP-labeled proteins with 488 nm laser excitation. Images were deconvolved with HUYGENS software (Huygens Professional version 21.10, Scientific Volume Imaging) to get a more precise signal for correlation. Focused ion beam (FIB) milling was performed with an Aquilos dual beam (FIB-SEM) microscope (Thermo Fisher Scientific). To locate the FIB milling position, we correlated the cryo-fluorescent images with the SEM images of the FIB machine by three-point correlation using the 3D-Correlation Toolbox (30). According to the correlative results, lamellae were milled at the indicated positions. For FIB milling, the tilt angles of the stage were set to 12° to 16°, and the focused ion beam operated at 30 kV. Furthermore, the beam currents were sequentially set to 0.3 nA at approximately 1 µm distance from the indicated positions, 0.1 nA at 800 nm, and 50 pA at 150 to 200 nm for lamellae polishing. The lamellae were stored in liquid nitrogen. The lamellae number and corresponding cell lines are listed in *SI Appendix*, Table S1.

### Cryo-ET Data Collection.

The cryo-ET tilt series were collected with a Titan Krios Microscope (Thermo Fischer Scientific) operated at a voltage of 300 KV and equipped with a quantum postcolumn energy filter and a K2 Summit direct electron detector (Gatan Inc). The tilt series were recorded from 68° to −52° (2° increment) starting at pretilts of 8° with the SerialEM software (45) and a dose-symmetric tilt-scheme (46). The recording magnifications were set at 33,000 with a pixel size of 4.39 Å, 42,000 with a pixel size of 3.52 Å, and 81,000 with a pixel size of 1.79 Å. The total dose was approximately 120 e^−^/Å^2^. The defocus ranged from −4.6 μm to −5.2 μm.

### Tomogram Reconstruction and Segmentation.

Tilt series were preprocessed with TOMOMAN software (https://github.com/williamnwan/TOMOMAN), performing beam-induced motion correction with MotionCor2 (47), tilt-series sorting, and contrast transfer function estimation with CTFFIND (48). The processed tilt series were aligned with IMOD (v4.10.49) using the patch-tracking method and reconstructed by weighted back-projection at binning 4 (49). The tomograms were denoised with cryo-CARE to increase contrast (50). The reconstructed tomograms and corresponding cell lines are listed in *SI Appendix*, Table S1.

Membrane segmentation was first detected with the TomoSegMemTV package (51) and analyzed using Amira software (Thermo Fisher Scientific). Microtubules were segmented with the convolutional neural networks of EMAN2 (52). For the annotation of ribosome positions, template matching was applied using the pyTOM toolbox (53). The ribosomes of HeLa cells were performed with a template of a human 80S ribosome structure (EMD-2938) (54) filtered to 40 Å, and the ribosomes of *Salmonella* with a template of an Escherichia coli 70S ribosome (EMD-2847) (55) were filtered to 40 Å. Structure visualization and movie generation were conducted with UCSF ChimeraX (56).

### Quantification and Statistical Analysis.

The membranes of the phagophore body and rim region were segmented. The rim region was calculated from the dilated region. The membrane was first segmented using the TomoSegMemTV package (membrane thickness factor set to 1). Then, Amira was used to refine the segmentation. Based on the segmented membrane, the intermembrane distance and the thickness of the phagophore double layers were calculated with the PyCurv software (57). The intermembrane distance and thickness distribution were plotted with Origin (OriginLab Corporation). Data were analyzed using the Mann–Whitney *U* test or two-sample *t* test. The length of stick-shaped proteins was measured with the IMOD measurement module (49). Membrane curvature was based on the segmented membrane and analyzed with PyCurv (radius hit: 10 nm, algorithm variants: augmented vector voting). The visualization was performed using ParaView (58).

## Supplementary Material

Appendix 01 (PDF)

Movie S1. Sequential slices back and forth through the representative tomogram in cross-section view and the 3D rendering models. The movie shows that the growing phagophore engulfing *Salmonella* establishes close interaction with the ER tube and the ER sheet at the two individual rim regions. The mCherry-GAL8 expressing HeLa cells were infected with GFP expressing *Salmonellae* and imaged at 1.5 hours p.i.. Related to Figure 2.

Movie S2. Sequential slices back and forth through the representative tomogram in cross-section view and the 3D rendering models. The movie shows that *Salmonella* is encapsulated by the growing phagophore and the matured autophagosome. The mCherry-GAL8 expressing HeLa cells were infected with GFP expressing *Salmonellae* and imaged at 2 hours p.i.. Related to *SI Appendix*, Figure S2C.

Movie S3. Enlarged sequential slices through the representative tomogram in cross-section view and the 3D rendering models. The movie shows that the growing phagophore rim connects with the ER tube via stick-shaped proteins. Scale bar represents 50 nm. Related to Figure 3A.

Movie S4. Enlarged sequential slices through the representative tomogram in cross-section view and the 3D rendering models. The movie shows that the growing phagophore rim connects with the ER tube/sheet via stick-shaped proteins. Scale bar represents 50 nm. Related to Figure 3C.

Movie S5. Enlarged sequential slices through the representative tomogram in cross-section view and the 3D rendering models. The movie shows membrane contact sites of a dilated phagophore rim with a ER tube and ER sheet bridged by macromolecule clusters. Scale bar represents 50 nm. The mCherry-GAL8 expressing HeLa cells were infected with GFP expressing *Salmonellae* and imaged at 1.5 hours p.i.. Related to Figure 4.

Movie S6. Sequential slices back and forth through the representative tomogram in cross-section view and the 3D rendering models. The movie shows that omegasome-like structures are found in close proximity to the ER. The mCherry-DFCP1 expressing HeLa cells were infected with GFP expressing *Salmonellae* and imaged at 2 hours p.i.. Related to Figure 5.

## Data Availability

Representative tomograms have been deposited at the Electron Microscopy Data Bank as entries: EMD-16417 (59) (Fig. 1*E*); EMD-16418 (60) (Fig. 1*F*); EMD-16419 (61) (Fig. 2*A*); EMD-16420 (62) (Fig. 2*D*); EMD-16421 (63) (Fig. 5*B*); EMD-16422 (64) (Fig. 4*A*).
