# Supplementary material for: In situ snapshots along a mammalian selective autophagy pathway
Source: Proc Natl Acad Sci U S A. 2023 Mar 14;120(12):e2221712120. doi: 10.1073/pnas.2221712120 (PMC10041112; doi:10.1073/pnas.2221712120)
Supplement: Supplementary file 1 — Appendix 01 (PDF) [file pnas.2221712120.sapp.pdf]

PNAS

Supplementary Information for

In situ snapshots along a mammalian selective autophagy pathway

Meijing Li[1,*], Ishita Tripathi Giesgen[2], Brenda Schulman[2], Wolfgang Baumeister[1,*] , and Florian Wilfling[1,3,*]

1, Max Planck Institute of Biochemistry, Department of Molecular Structural Biology, Am Klopferspitz 18, 82152 Martinsried, Germany
2, Max Planck Institute of Biochemistry, Department of Molecular Machines and Signaling, Am Klopferspitz 18, 82152 Martinsried, Germany
3, Max Planck Institute of Biophysics, Mechanisms of Cellular Quality Control, 60438 Frankfurt a. M., Germany

* Corresponding Authors: meijing@biochem.mpg.de
baumeist@biochem.mpg.de
florian.wilfling@biophys.mpg.de

This PDF file includes:

Figures S1 to S5
Legends for Movies S1 to S6
SI References
Table S1

Other supplementary materials for this manuscript include the following:

Movies S1 to S6

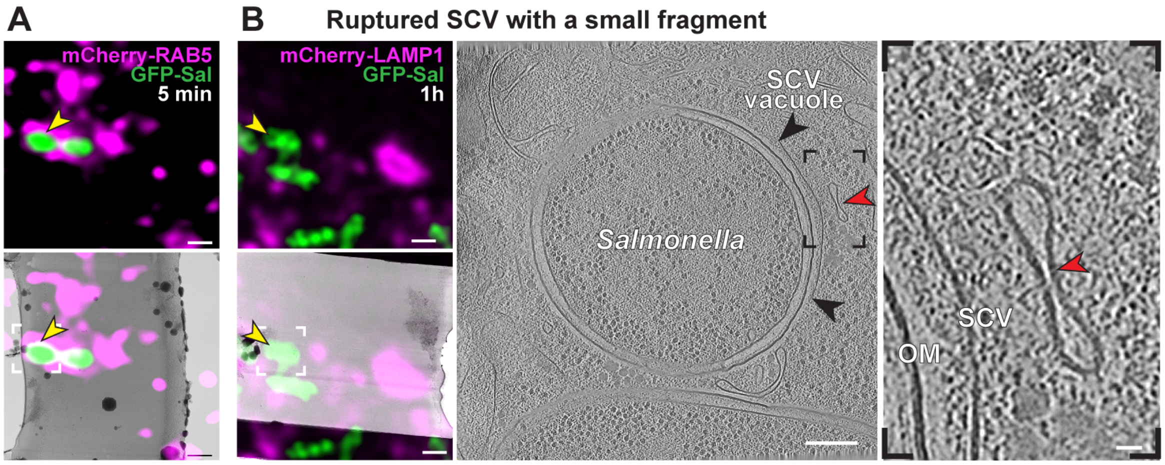

Fig. S1. Characterization of SCV damage by correlative cryo-ET. (A) Cryo-fluorescent image and the corresponding lamella overlay of Figure 1C. **(B)** mCherry-LAMP1 expressing HeLa cells were infected with GFP-expressing *Salmonellae*. Tomographic slices show *Salmonella* in an almost completely ruptured SCV (black arrowheads), which is targeted by an early phagophore (red arrowhead). Scale bars represent 1 µm in (A) and the left panel of (B), 200 nm in the middle panel of (B), 20 nm in the right panel of (B).

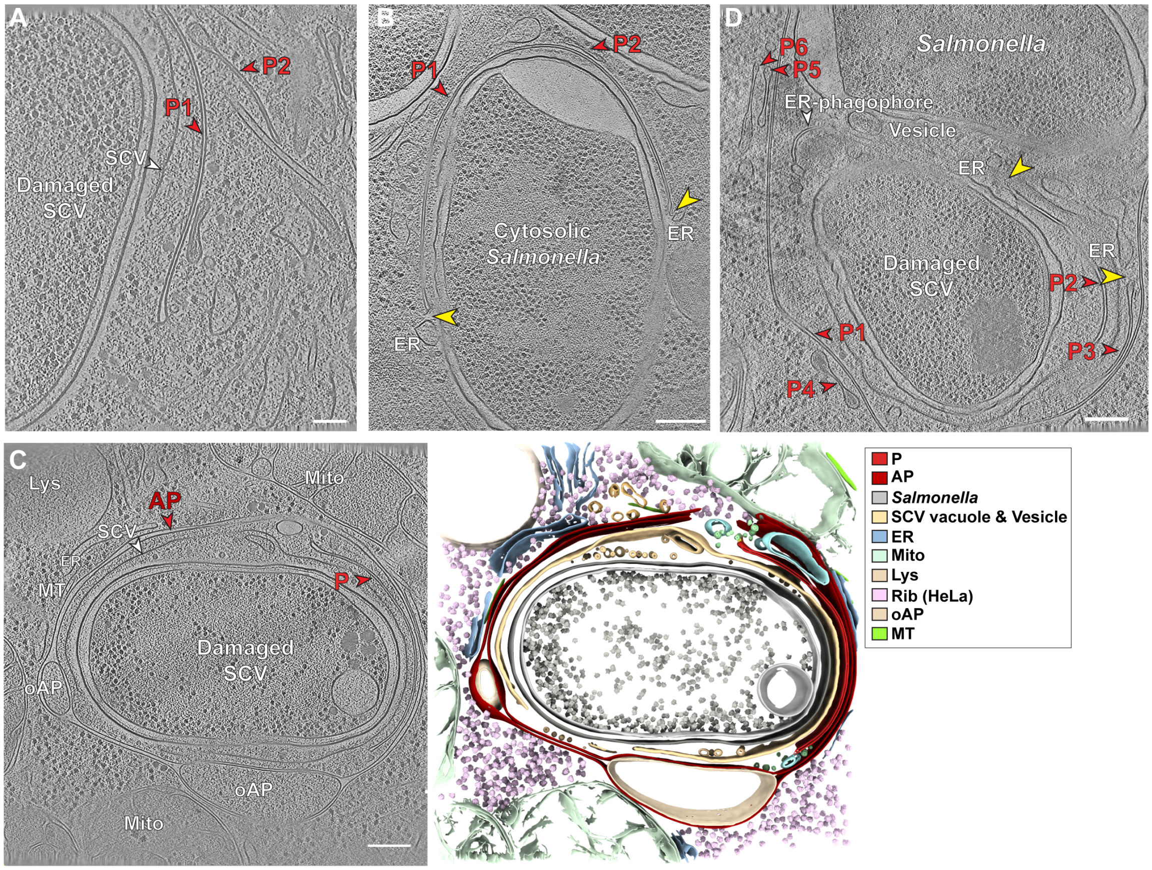

Fig. S2. Visualization of multiple phagophores around the damaged SCV or cytosolic *Salmonella*. **(A)** Tomographic slice showing a highly dilated phagophore rim. 19 out of 75 rims showed such a phenotype. **(B)** Tomographic slice showing an expanded phagophore and an early phagophore at a cytosolically exposed *Salmonella*. Yellow arrows indicate membrane contact sites between the phagophore and the ER. **(C)** Tomographic slice showing a potentially closed autophagosome enwrapping a *Salmonella* and a phagophore. The right panel shows the 3D rendering model. oAP, other autophagosome. **(D)** Tomographic slice showing multiple phagophores enclosing two *Salmonellae*. The giant phagophore (P1) enwraps unselectively ER and vesicles, and an ER-phagophore. The mCherry-Gal8 expressing HeLa cells were infected with GFP expressing *Salmonellae* at 2 hours p.i.. Scale bars represent 100 nm in (A) and 200 nm in (B, C, D).

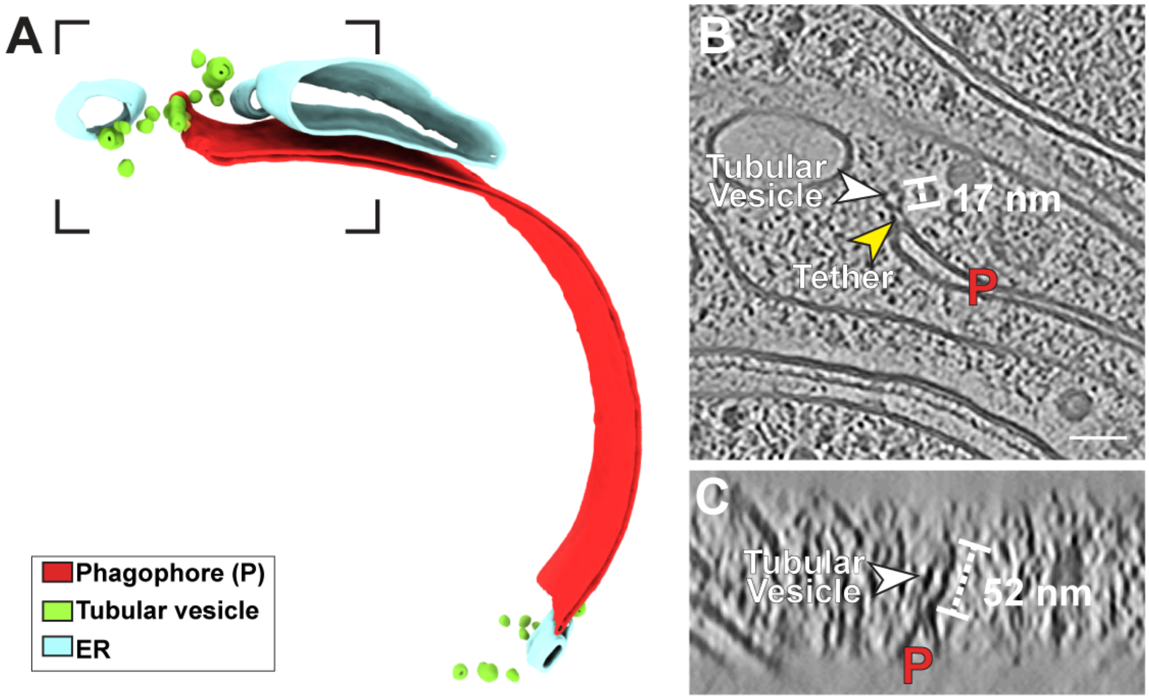

Fig. S3. A thin tubular vesicle is connected with the phagophore rim via stick-shaped densities. (A) 3D rendering model of the phagophore and the associating vesicles in *SI Appendix* Figure 2C. **(B)** The tomographic slice shows a tubular vesicle connected with the phagophore rim via stick-shaped densities. The scale bar represents 50 nm. **(C)** The XZ view tomographic slice shows that the vesicle is a thin tubular vesicle.

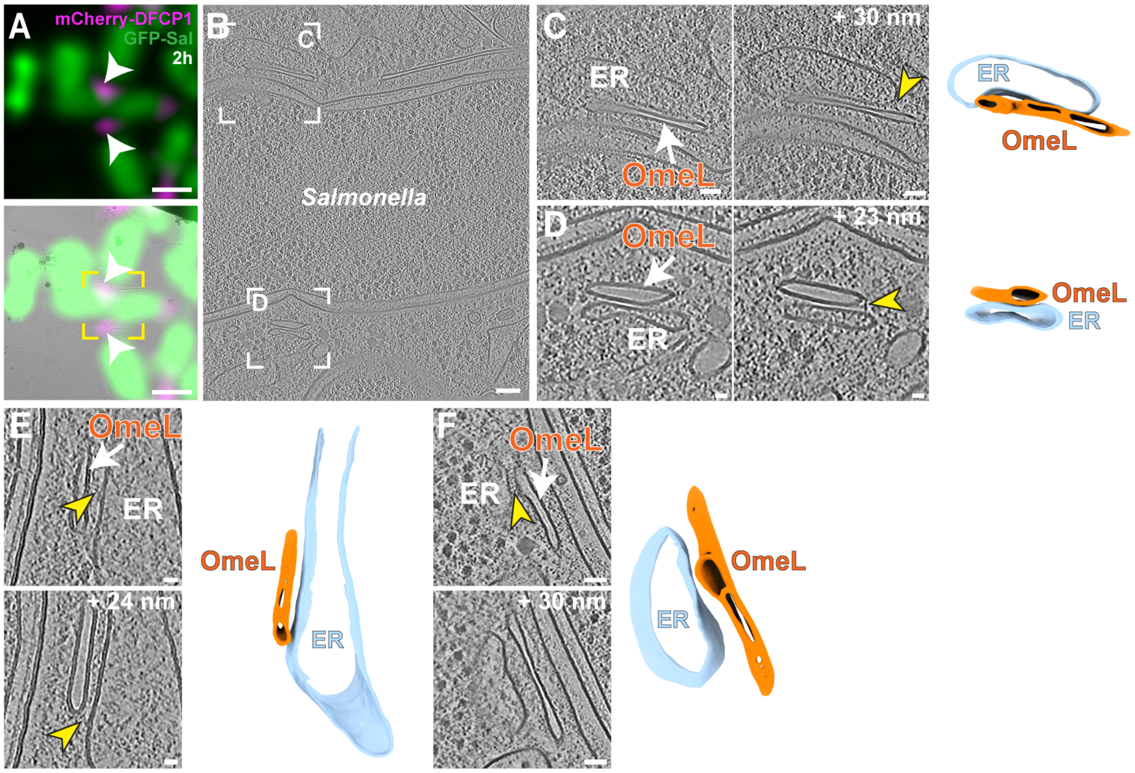

Fig. S4. Disc-shaped omegasome-like structures are closely associated with the ER. (A) Cryo-fluorescent image and the corresponding lamella overlay show two positions with mCherry-DFCP1 signal around *Salmonella*. The mCherry-DFCP1 expressing HeLa cells were imaged at 2 hours p.i.. White arrowheads indicate the targeted DFCP1 signals. The white box indicates the cryo-ET tilt series acquisition area. **(B)** The tomographic slice of (A) shows two disc-shaped omegasome-like structures around *Salmonella*. **(C and D)** Enlarged sequential tomographic slices and 3D rendering models highlight the tight apposition of the OmeL and the ER, as well as membrane contact sites (indicated by yellow arrowheads). **(E and F)** Additional, examples of disc-shaped omegasome-like structures and their contact with the proximal ER. Yellow arrowheads indicate the protein densities at membrane contact sites. The scale bars represent 1 μm in (A), 100 nm in (B), 50nm in (E), and 20 nm in (C, D, F).

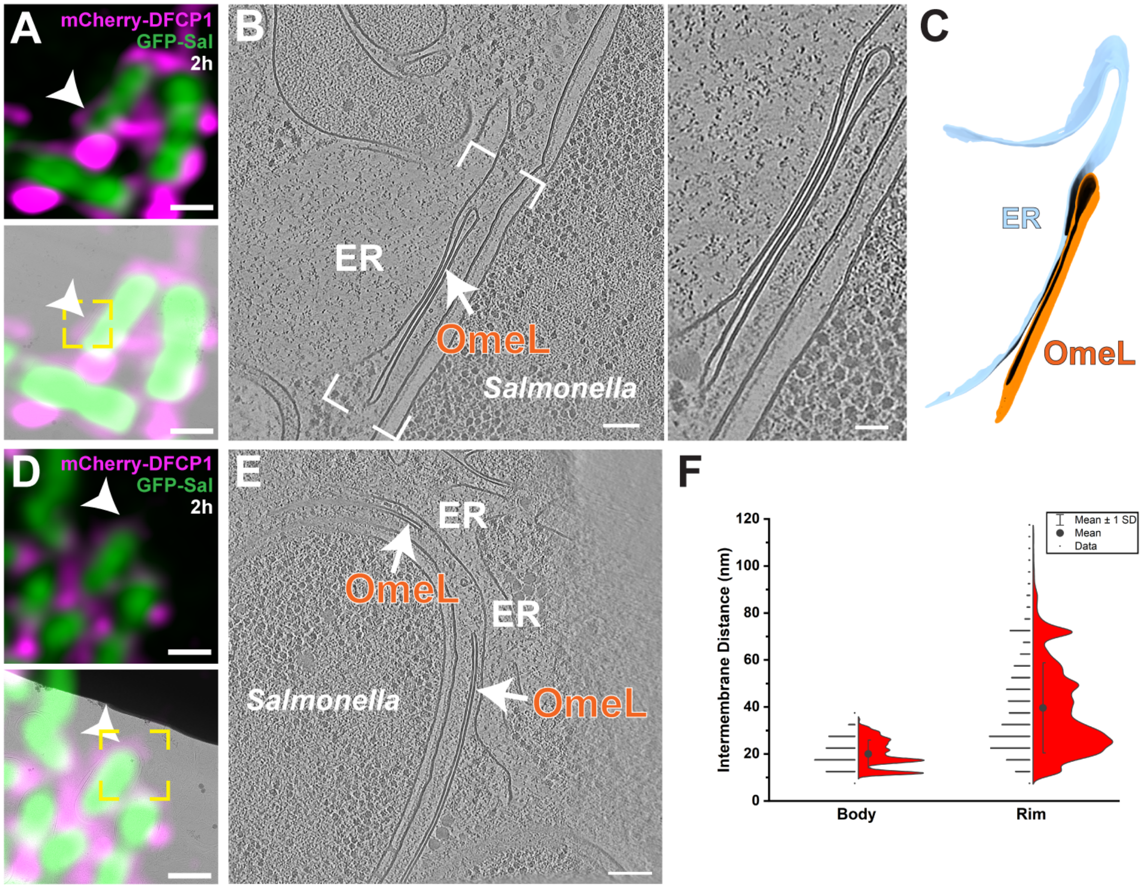

Fig. S5. Analysis of expanded omegasome-like structures. (A and D) Cryo-fluorescent image and the corresponding lamella overlay show two positions with mCherry-DFCP1 signal around *Salmonella*. The mCherry-DFCP1 expressing HeLa cells were imaged at 2 hours p.i.. White arrowheads indicate the targeted DFCP1 signals. The white box indicates the cryo-ET tilt series acquisition area. **(B)** Corresponding tomographic slices of (A) show an expanded phagophore in close proximity to the ER. **(C)** 3D rendering model of the expanded phagophore and the ER. **(E)** The corresponding tomographic slice of (D) shows two expanded phagophores. **(F)** Violin plot showing the distribution of the intermembrane distance of the body region and rim region of expanded omegasome-like structures (N = 12). The average intermembrane distance is 20.0 ± 5.9 nm and 39.6 ± 19.2 nm, respectively. The scale bars represent 1 μm in (A, D), 100 nm in (B, E), and 50 nm in the right panel of (B).

Table S1. Summary of cell lines and the number of corresponding tomograms.

Cell line	Salmonella infection time	Number of Lamellae	Number of tomograms	Number of phagophore structures
mCherry-Rab5B	5 - 30 mins	16	12	1
mCherry-Galectin8	1 -2.5 hours	58	62	68
LAMP1-mCherry	1 hour	11	12	14
eGFP-LC3B	1.5 – 2.5 hours	11	11	5
mCherry-DFCP1	2 hours	21	25	28

Movie S1. Sequential slices back and forth through the representative tomogram in cross-section view and the 3D rendering models. The movie shows that the growing phagophore engulfing *Salmonella* establishes close interaction with the ER tube and the ER sheet at the two individual rim regions. The mCherry-GAL8 expressing HeLa cells were infected with GFP expressing *Salmonellae* and imaged at 1.5 hours p.i.. Related to Figure 2.

Movie S2. Sequential slices back and forth through the representative tomogram in cross-section view and the 3D rendering models. The movie shows that *Salmonella* is encapsulated by the growing phagophore and the matured autophagosome. The mCherry-GAL8 expressing HeLa cells were infected with GFP expressing *Salmonellae* and imaged at 2 hours p.i.. Related to *SI Appendix*, Figure S2C.

Movie S3. Enlarged sequential slices through the representative tomogram in cross-section view and the 3D rendering models. The movie shows that the growing phagophore rim connects with the ER tube via stick-shaped proteins. Scale bar represents 50 nm. Related to Figure 3A.

Movie S4. Enlarged sequential slices through the representative tomogram in cross-section view and the 3D rendering models. The movie shows that the growing phagophore rim connects with the ER tube/sheet via stick-shaped proteins. Scale bar represents 50 nm. Related to Figure 3C.

Movie S5. Enlarged sequential slices through the representative tomogram in cross-section view and the 3D rendering models. The movie shows membrane contact sites of a dilated phagophore rim with a ER tube and ER sheet bridged by macromolecule clusters. Scale bar represents 50 nm. The mCherry-GAL8 expressing HeLa cells were infected with GFP expressing *Salmonellae* and imaged at 1.5 hours p.i.. Related to Figure 4.

Movie S6. Sequential slices back and forth through the representative tomogram in cross-section view and the 3D rendering models. The movie shows that omegasome-like structures are found in close proximity to the ER. The mCherry-DFCP1 expressing HeLa cells were infected with GFP expressing *Salmonellae* and imaged at 2 hours p.i.. Related to Figure 5.

References

1. D. Zeuschner *et al.*, Immuno-electron tomography of ER exit sites reveals the existence of free COPII-coated transport carriers. *Nat Cell Biol* **8**, 377-383 (2006).
2. Y. S. Bykov *et al.*, The structure of the COPI coat determined within the cell. *elife* **6** (2017).
3. C. Appenzeller-Herzog, H. P. Hauri, The ER-Golgi intermediate compartment (ERGIC): in search of its identity and function. *J Cell Sci* **119**, 2173-2183 (2006).
